# Supplementary material for: The relationship between NLR, LDL-C/HDL-C, NHR and coronary artery disease
Source: PLoS One. 2024 Jul 10;19(7):e0290805. doi: 10.1371/journal.pone.0290805 (PMC11236180; doi:10.1371/journal.pone.0290805)
Supplement: Attachment — Submitted filename: Response to Reviewers.doc [file pone.0290805.s002.doc]

**About Journal Requirements:**

1.My manuscript has been modified according to the style requirements of PLOS ONE, including file naming requirements.

2.About“https://www.science.gov/topicpages/m/macrophage+cholesterol+homeostasis”,the mention of "macrophage cholesterol homeostasis" in this article confirms that there was no plagiarism. The content of this article can be referred to in the references, and the content cited in this article is not related to "macrophage cholesterol homeostasis".

About “https://bmccardiovascdisord.biomedcentral.com/articles/10.1186/s12872-020-01771-z”,t

he above article only mentioned the ratio of neutrophils to HDL-C. This article cites a combination of inflammation and lipid metabolism, with more data cited and more convincing results. There is no plagiarism in this article and there is no interest in it.

And some duplicated texts have been rephrased according to the requirements of the editorial department.

3.When I resubmitted, I had already provided the correct grant numbers in the "Funding Information" section.

4.ORCID iD has been provided as required and validated in Editorial Manager.

**About Additional Editor Comments:**

The statistical methods have been modified and the manuscript has been revised.

**About**

**1.Is the manuscript technically sound, and do the data support the conclusions?**

The manuscript has been revised as required to align with scientific research and draw conclusions based on experimental data.

**2.Has the statistical analysis been performed appropriately and rigorously?**

A detailed description of the statistical analysis of the article has been provided to ensure its appropriateness and rigor.

**3.Have the authors made all data underlying the findings in their manuscript fully available?**

This article provides all the data found in the manuscript.

**4.Is the manuscript presented in an intelligible fashion and written in standard English?**

The manuscript is presented in an understandable manner and written in standard English.

**About Reviewer #1:**

The selection criteria for the article have been modified by adding "or" between the three criteria.

1.A detailed description of the statistical analysis of the article has been provided to ensure its appropriateness and rigor.Kolmogorov–Smirnov test was used to verify that the continuous variable was normally distributed.

2.The strength of correlation has been emphasized in the analysis as required.

**About Reviewer #2:**

1.All abbreviations in the text have been explained as required.

2.Table 1- All data were tested for normality using the Kolmogorov Smirnov test method.

3.The name of the Gensini score has been modified in the table.

4.Propose multivariate analysis based on previously published articles to enable the application of more data and improve the accuracy of the results.

**About PLOS authors have the option to publish the peer review history of their article (what does this mean?). If published, this will include your full peer review and any attached files.**

No
