# Supplementary material for: The relationship between NLR, LDL-C/HDL-C, NHR and coronary artery disease
Source: PLoS One. 2024 Jul 10;19(7):e0290805. doi: 10.1371/journal.pone.0290805 (PMC11236180; doi:10.1371/journal.pone.0290805)
Supplement: Attachment — Submitted filename: Response to Reviewers.doc [file pone.0290805.s003.doc]

**About Journal Requirements:**

1.I have checked my reference list to ensure it is complete and correct.

**About Additional Editor Comments:**

1.The title in the submission system has been modified, and the work done by the author in the title has been deleted.

2.I have had native English speakers edit the main text.

**Special instructions：**

Explanation on Pailing Xie's Change to Co corresponding Author：The author has undertaken many important tasks in this article and participated in Conceived and designed the experiments、Performed the experiments、Contributed reagents, materials, analysis tools or data、Writing – original draft、Writing – review & editing and main text English edited.
