# Supplementary material for: The relationship between NLR, LDL-C/HDL-C, NHR and coronary artery disease
Source: PLoS One. 2024 Jul 10;19(7):e0290805. doi: 10.1371/journal.pone.0290805 (PMC11236180; doi:10.1371/journal.pone.0290805)
Supplement: Attachment — Submitted filename: Response to Reviewers.doc [file pone.0290805.s004.doc]

**We have asked the native English speaker to help us check the grammarly and the clarity of the manuscript.**
